# Controlling shareholders’ equity pledge and corporate innovation investment—empirical analysis based on pharmaceutical manufacturing

**DOI:** 10.3389/fpubh.2024.1478335

**Published:** 2025-01-03

**Authors:** Jiawen Li, Xingyu Zhao, Su Wang, Yuwen Chen

**Affiliations:** ^1^School of Business Administration, Shenyang Pharmaceutical University, Shenyang, China; ^2^Drug Regulatory Research Base of NMPA, Research Institute of Drug Regulatory Science, Shenyang Pharmaceutical University, Shenyang, China

**Keywords:** equity pledges, innovation, equity incentives, financing constraints, pharmaceutical manufacturing industry

## Abstract

Enterprise innovation investment is influenced by the actions of innovation subjects, whereas regulating shareholders’ equity pledge behavior facilitates innovation investment and finance but also carries dangers and affects enterprise innovation investment. Methods:This paper builds an unbalanced panel model to empirically analyze the impact of controlling shareholders’ equity pledges on corporate innovation and its heterogeneous characteristics. It also looks at the moderating role of corporate financing constraints and the mediating role of equity incentives, using data from A-share listed companies in China’s pharmaceutical manufacturing industry from 2015 to 2022. Innovation investment is substantially inversely correlated with controlling shareholders’ equity pledge; that is, firms’ creative behavior and intensity are inhibited by equity pledge. Results and conclusions:The results also show that controlling shareholders’ equity commitments have a more pronounced negative impact on enterprises’ ability to innovate than non-state-owned and decentralized equity firms. The relationship between company innovation and the equity pledge of controlling shareholders is somewhat mediated by equity incentives. The relationship between controlling shareholders’ equity promises and enterprises’ innovation is negatively moderated by financing limitations, which also reduces R&D expenditure and stifles innovation.

## 1 Introduction

China is currently going through a significant phase of upgrading its economic structure and changing its driving forces. To support high-quality economic development, the country must move away from factor-driven and scale-driven economic development and toward innovation-driven economic development. But while being the driving force behind independent innovation, China’s businesses continue to struggle with poor R&D expenditure and low desire. The question of how to encourage business innovation has therefore gained a lot of attention in scholarly research in recent years.

The act of pledging a pledgor’s company equity to meet financial needs and secure funding is known as an equity pledge, or pledge of rights (1). Prior to 2013, the equity pledge (EP) business in China saw limited growth. However, since the introduction of the EP buyback trading mechanism, securities firms have been eligible to transact EPs. Securities businesses typically avoid transfer and transaction taxes, resulting in faster and more efficient EP transactions compared to other financial institutions.

China’s listed companies have two options when it comes to funding constraints: bank loans or equity pledge financing. On the other hand, bank loans are slow to disburse, contain a lot of restrictive terms, and complex procedures (2). Stocks, on the other hand, are very liquid, freely priced, highly transparent, easily accepted by the market, have relatively easy procedures, allow for faster financing, and both outstanding and restricted shares can be pledged for funding, with no effect on pledged shares’ voting rights. Thus, equity pledges have steadily become a popular financing strategy in the capital market (3). According to a report provided by China Securities Depository & Clearing Corporation, large shareholders pledged equity in 2,447 listed businesses in 2019, with more than half of shares pledged and 33 companies pledging more than 60% of their shares. However, the equity promise behavior of dominant owners poses specific hazards. When the pledgor makes a loan to the pledged listed firm, three indications are typically set: the pledge rate, warning line, and closure line. When the value of pledged shares goes below the warning line, shareholders must replace their promises to ensure that the residual value does not exceed the entire value of existing lending and pledges (4). The early warning line corresponds to the final red line of the pledge; if shareholders are unable to repay the loan to redeem the stock or if the stock’s value falls below the early warning line, the pledgee may liquidate the stock to protect their interests and mitigate losses promptly. It is important to recognize that while equity pledging can facilitate financing for corporate innovation, the necessity to realize share prices poses a risk. If the controlling shareholder fails to timely release the pledge or remit the deposit, they may forfeit control over the listed enterprise, thereby exposing it to significant risk. To prevent a loss of control, principal owners will implement various market value management strategies to stabilize and enhance the share price (5). Therefore, do dominant owners maintain their position of power by discouraging creative actions that carry a higher degree of risk and profit uncertainty? Do resources play a moderating role in the relationship?

Moreover, controlling shareholders typically do not engage directly in the operational aspects of the company; rather, their impact on the company’s activities is exerted through the board of directors, which in turn shapes the conduct of the company’s executives. In accordance with principal-agent theory, controlling shareholders, acting as the principal, are able to establish suitable incentives for corporate executives, who serve as the agent. This alignment ensures that the interests of the executives coincide with those of the shareholders, thereby motivating the executives to prioritize the shareholders’ interests in their actions (6). Consequently, the controlling shareholders seek to preserve the stability of the company’s existing share price, endeavoring to align the interests of the company’s executives with the maintenance of this stability in a manner that is deemed more advantageous. The motivations of executives within the organization are predominantly associated with their remuneration and incentives. Controlling shareholders with equity pledges are likely to influence the incentives of company executives via the board of directors, aiming to enhance their alignment with the stability of the company’s short- to medium-term share price. This creates an incentive for executives to implement measures that effectively maintain share price stability in the short- to medium-term (7).

The pharmaceutical manufacturing industry in China differs significantly from other industries in terms of industry status, technological innovation, business systems, and other factors. The research and development of new pharmaceuticals is characterized by high investment, high output, high risk, and a long cycle, which is due to the complexity of the R&D process and the combined impact of a number of factors. Drug R&D not only takes a significant commitment of funds and effort, but it also carries a high risk of failure. However, the market return after success is equally significant (8). High risk is primarily manifested in the following dimensions: Firstly, the high technical complexity of new drug research and development necessitates overcoming numerous scientific and technical challenges. Secondly, the stringent regulatory approval process mandates that drugs undergo rigorous scrutiny prior to market release. Thirdly, significant market uncertainty persists, as even successful drug development may face unpredictable acceptance and competition. The large output is attributable to the substantial market returns associated with successful drug development. Upon achieving market acceptability, a novel medicine can generate substantial revenues while also providing enduring competitive benefits and brand influence for pharmaceutical companies. Consequently, the innovation and enhancement of the pharmaceutical manufacturing sector are crucial for elevating China’s pharmaceutical business on the global stage and for protecting public health.

In light of the aforementioned factors, this paper employs the unbalanced panel data of China’s A-share listed companies in the pharmaceutical manufacturing industry from 2015 to 2022 as the research background. The research demonstrates that the innovation investment of firms is impeded by the control of shareholders’ equity pledges. Equity incentives serve as a partial intermediary in the process of controlling shareholders’ equity pledges, and financing constraints serve as a moderating factor in the process of controlling shareholders’ equity pledges.

## 2 Literature review and research hypotheses

### 2.1 Controlling shareholders’ equity pledge and corporate innovation investment

#### 2.1.1 Mediating effects of equity incentives

Currently, there is some debate regarding the influence of controlling shareholders’ equity pledges on the innovation investment of enterprises. First and foremost, the equity pledge behavior of controlling shareholders has the potential to encourage corporate innovation investment. This is because when controlling shareholders invest the pledged funds in innovative projects, it will enhance corporate innovation investment. Additionally, controlling shareholders’ equity pledge behavior introduces external supervision from banks and other financial institutions, which acts as a form of external pressure to encourage the enterprise to increase its innovation investment. This process also promotes the sustainable development of the enterprise (9). Additionally, Lu Rui discovered that the company’s operating conditions are considerably enhanced and its sound development is promoted by the close supervision of pledge parties, such as banks and credit companies (10). The data of A-share listed companies in the pharmaceutical manufacturing industry from 2012 to 2017 was analyzed by Yan Fulei and Shi Yiming using a fixed-effects model. The results indicated that controlling shareholders’ equity pledges significantly promoted corporate innovation investment, and the promotional effect was more pronounced as the proportion of equity pledges increased (11). Lin Yan observed that banks and other financial institutions establish warning lines, closing lines, and other standards. In order to avoid being included in the guarantee, controlling shareholders must actively enhance the company’s operating conditions, increase market sales profits, and meet or exceed the anticipated objectives (12). Tan Yan and Wu Jing demonstrated that the interests of the two parties are interconnected as a result of the pledge of equity. SMEs will strive to enhance the company’s operating conditions and sales performance in order to efficiently utilize the loan, increase market share and profits, and promptly reclaim pledged shares (13).

Equity pledges may inhibit firms’ investment in innovation. Following equity pledges, controlling shareholders often reserve funds to mitigate the risk of share price collapse and control transfer, resulting in a “crowding out” effect on innovation activities. Cai H indicated that, from the perspective of firms’ risk-taking levels, controlling shareholders’ equity pledges alter the risk of their investment portfolios and diminish the firms’ risk-taking capacity, thereby hindering investment in innovation (14). Aghion observed that shareholders prioritize the margin of safety in their investments over the potential return, leading to a more conservative approach in their investment decisions (15). Some scholars have observed that controlling shareholders may utilize their influence to minimize investments in high-risk projects to safeguard their control (16, 17). Zhai Shiyun indicated that controlling shareholders, motivated by control protection, might opt to retain sufficient cash reserves to address potential pledge-related issues, even when the expected returns from innovative projects exceed their cost of capital. This behavior is particularly evident when share prices approach critical thresholds, leading to a preference for avoiding investments in capital-intensive innovative projects. They may opt to replenish or release the pledge, retaining sufficient cash and avoiding investment in innovative projects that require significant capital (18). Chen Xiaopeng and Jiang Shaobo argue that, from the standpoint of financing constraints, the equity pledge behavior of controlling shareholders signals to the capital market that enterprises are experiencing significant financing limitations. This perception exacerbates the financing environment for these enterprises and hampers the advancement of their innovative projects (19). Based on the above discussion, the following hypotheses are proposed:

*H1*: The equity pledges of controlling shareholders negatively influence the innovation levels within firms.

### 2.2 Mediating effects of equity incentives

According to principal-agent theory, information asymmetry and divergent interests exist between corporate shareholders and executives. This misalignment may lead executives to prioritize personal gain over the company’s welfare, resulting in moral hazard and adverse selection. Corporate innovation entails significant risk and uncertainty, leading executives to often eschew such risks in order to safeguard their own interests. Research indicates that equity incentives serve as an effective mechanism to align executives’ preferences with long-term corporate objectives, thereby facilitating the pursuit of maximized long-term corporate value. Chen Jinyong discovered that listed companies with management shareholding tend to increase R&D investment, which subsequently enhances the innovation efficiency of the enterprise to a certain degree (20). Furthermore, according to the hierarchy of needs theory, offering equity incentives to executives can help them feel more a part of the company and meet their higher-level needs. This can encourage executives to conduct innovative R&D and to share the long-term benefits that innovation has for the business. The equity pledge by controlling shareholders may diminish the enterprise’s R&D investment; however, the shareholding executives also fulfill a supervisory governance function. Controlling shareholders, often not engaged in daily operations, may exhibit opportunistic behavior that adversely affects small and medium-sized shareholders, necessitating the complicity of specific executives. To safeguard their future interests, executives may refrain from cooperating with controlling shareholders and oppose specific opportunistic behaviors (21). Based on the above analysis, the following hypotheses are proposed:

*H2*: Providing equity incentives to executives may mitigate the adverse relationship between equity pledges by controlling shareholders and investment in innovation.

### 2.3 The moderating role of financing constraints

Controlling shareholders’ equity pledges transmit negative signals, such as financing constraints, to both internal and external stakeholders. This communication can result in a decline in share price, thereby intensifying the financing constraints faced by the enterprise. Furthermore, following the equity pledge, market value management is typically implemented to mitigate the risk of control transfer. However, this practice tends to exacerbate information asymmetry both internally and externally, thereby heightening the financing constraints faced by the enterprise. The impact of financing constraints on enterprise innovation can be addressed through endogenous channels, as well as debt and equity financing options. The financing priority theory posits that enterprises prioritize funding sources in the following sequence: endogenous financing, debt financing, and equity financing (22). Previous research indicates that many enterprises face challenges related to insufficient endogenous financing, highlighting the significance of exogenous financing channels. Breakthrough innovation is characterized by extended lead times, significant risk, and uncertain returns; thus, the conflict of interest between shareholders and creditors may hinder such innovation. Enterprises with limited financial resources often prefer debt financing to support breakthrough innovation; however, creditors typically oppose the allocation of these funds for such activities. The creditor’s return remains fixed, independent of the project’s success, and does not increase with the associated risk. In the event of project failure, the creditor risks losing the entire principal amount, presenting a significant risk. Consequently, the creditor may decline to provide funding if the bondholder is not adequately compensated for this risk. The company’s funds will become constrained, necessitating a reduction in investment in R&D activities (23). From the firm’s perspective, however, it is challenging to secure funding for innovative breakthroughs when dealing with a high degree of external financing constraints; this is due to the fact that banks and other financial institutions are understandably wary of lending money to businesses that have a history of trouble repaying loans, which compounds the difficulty of securing funding overall. There may be an imbalance of knowledge both inside and outside the company as a result of companies’ efforts to keep their secrets a secret. Because of this, the cost of obtaining external finance may rise, which in turn may decrease the incentive for businesses to undertake innovative, game-changing projects (24). This raises the cost of external finance, which decreases the motivation for breakthrough innovative activities.

*H3*: Equity promises can have a negative effect on corporate innovation due in large part to financing constraint behavior.

## 3 Study design

### 3.1 Sample and data sources

Before 2013, the growth of the equity pledge industry in China was comparatively modest and has been a very convoluted process, so this paper takes A-share listed companies in the pharmaceutical manufacturing industry from 2015 to 2022 as the original sample. In order to ensure the accuracy and completeness of the research conclusions, the original samples in this paper are processed as follows: (1) ST companies are excluded. Because generally ST listed companies apply to bankruptcy accounting standards and are not comparable with listed companies in other industries, they are eliminated. (2) Delete the listed companies with missing data and those with less than 20 industry sample sizes; (3) Delete the companies that have been specially treated in the current year, and this paper shrinks the tail at the front and back ends of the continuous variables. The raw data of controlling shareholders’ equity pledge, corporate innovation investment and other variables are from CSMAR database, and Stata17.0 is used to test the data model.

### 3.2 Definition of variables

#### 3.2.1 Dependent variable

Corporate innovation investment (RD): this paper draws on the study of Li Changqing (25) and chooses the intensity of R&D investment to reflect the innovation investment of listed companies, i.e., total R&D investment as a percentage of operating revenue is used to measure the innovation investment of enterprises.

#### 3.2.2 Independent variable

Controlling shareholders’ shareholding proportion (Ple): with reference to the study by Zhu Lei (26), this paper chooses to measure the number of shares not yet unlocked by controlling shareholders at the end of the year as a proportion of the total number of shares held.

#### 3.2.3 Intermediary variable

Equity incentives (mo): According to Miao’s research, a listed company’s shares held by all of its executives are calculated as a percentage of the firm’s total shares (27).

#### 3.2.4 Moderator variable

Financing constraints (kz): this paper draws on Hadlock and Pierce (28). The kz index is constructed to measure the degree of corporate financing constraints through five indicators: total assets, interest coverage multiple, gearing ratio, operating cash flow and cash holding level. First of all, the above financial indicators are divided according to the annual industry median, when the enterprise’s total assets, interest coverage multiple, operating cash flow and cash holding level is lower than the industry median, it should be set to 1, otherwise it should be set to 0.

If the gearing ratio exceeds the industry average, it is set to 1, otherwise it is set to 0. On this basis, the five indicators are summed up to obtain the kz score, and the Order e d Lo gi t model is used to implement the regression, and the regression coefficients are used to calculate the kz index of each listed company. The higher the value of the kz index, the greater the degree of financing constraints suffered by the enterprise.

#### 3.2.5 Control variables

This paper sets firm size (Size), firm age (Age), gearing ratio (Lev), profitability (Roa) and firm growth (Growth) as control variables. In addition to this, year dummy variables and province dummy variables are included in this paper in order to control the effect of year and industry. The definitions of the relevant variables are shown in Table 1.

**Table 1:** List of variable definitions.

Variable type	Variable name	Variable symbol	Variable meaning
Explanatory variable	Level of innovation inputs	rd	Total R&D expenditures/operating income
Explanatory variable	Controlling shareholders’ equity weight of pledges	ple	Total number of equity pledges by controlling shareholders/total number of shares held by the enterprise at the end of the current period
Intermediary variable	Equity incentive	Mo	Total number of shares held by all executives of a listed company/total number of shares of that company ratio
Moderator variable	Financing constraints	Kz	See calculation above
Control variable	Age of business	age	Number of years the business has been established
	Enterprise size	size	The total assets of the enterprise at the end of the period are taken as the natural logarithm.
	Profitability	roa	Net profit/total assets of the enterprise at the end of the period
	Corporate debt ratio	lev	Total liabilities/total assets of the enterprise at the end of the period
	Growth capacity	growth	(Current incidence of operating income—Previous incidence of operating income)/Previous incidence of operating income

### 3.3 Model construction

#### 3.3.1 Test model of the impact of controlling shareholders’ equity pledge on firms’ innovation investment

In order to test the inhibitory effect of controlling shareholders’ equity pledge on corporate innovation investment, multiple regression model (1) is constructed to verify H1 and H2. The specific model is shown below:


(1) $$RD_{i,t}=\alpha 0+\alpha 1\mathrm{Pl}e_{i,t}+\mathrm{contro}l_{i,t}+\sum \mathrm{Year}+\sum \mathrm{Pro}+\varepsilon_{i,t}$$

In Equation 1, RD_i,t_ is an explanatory variable indicating the level of firms’ innovation investment, Ple_i,t_ is the explanatory variable of this paper controlling shareholders’ equity pledge, and Control_i,t_ is a series of control variable coefficients. In addition, the fixed effects of province and year are also controlled. α1 represents the impact of controlling shareholders’ equity pledge on firms’ innovation input. If α1 is significantly negative, it indicates that the behavior of controlling shareholders’ equity pledge will have a dampening effect on firms’ innovation investment, which is not conducive to the development of innovation activities, and H1 is verified.

#### 3.3.2 Test model for the mediating effect of equity incentives


(2) $$MO_{i,t}=\beta 0+\beta 1\mathrm{Pl}e_{i,t}+\mathrm{contro}l_{i,t}+\varepsilon_{i,t}$$

(3) $$RD_{i,t}=\gamma 0+\gamma 1\mathrm{Pl}e_{i,t}+\gamma 2MO_{i,t}+\mathrm{contro}l_{i,t}+\varepsilon_{i,t}$$

In order to test the risk transmission mechanism of equity incentives in the process of controlling shareholders’ equity pledges inhibiting firms’ innovation inputs, this paper draws on the Wen Zhonglin (29) mediated effect model. On the basis of the previous H1 is established, the regression of the following models (2, 3) is carried out. If the coefficients α1, β1, γ2 and γ1 are significant, equity incentives play a partial mediating role and H2 holds, otherwise, equity incentives play a full mediating effect.

#### 3.3.3 Test model for the moderating effect of financing constraints

In order to verify the moderating effect of financing constraints, this paper constructs a moderating effect model, i.e., adding the interaction term (kz × Ple) between financing constraints and controlling shareholders’ equity pledges in model (1), and if the coefficient of the interaction term is significantly positive, then H3 is established. Drawing on the moderating effect model of Wen Zhonglin (30), in order to avoid the covariance effect of the existence of multiple variables, centering is carried out before the interaction, and the model Equation 4, is constructed as follows:


(4) $$\begin{array}{l} RD_{i,t}=\alpha 0+\alpha 1\mathrm{Pl}e_{i,t}+\alpha 2kz_{i,t}+\alpha 3\mathrm{Pl}e_{i,t}\\ {}*kz_{i,t}+\mathrm{contro}l_{i,t}+\sum \mathrm{Year}+\sum \mathrm{Pro}+\varepsilon_{i,t} \end{array}$$

## 4 Results of empirical analysis

### 4.1 Descriptive statistics

Table 2 shows the descriptive statistics of the main variables in this paper, the results show that: from the explanatory variables, it can be seen that listed companies in the pharmaceutical manufacturing industry have innovative behaviors to varying degrees, the standard deviation of the innovation investment index is 5.423, the maximum and minimum values are 23.14 and 1.28 respectively, indicating that there are large differences in the degree of innovation investment in different listed companies. The mean value of the core explanatory variable controlling shareholders’ equity pledge degree (Ple) is 9.367, and the standard deviation is 9.147, i.e., 93.67% of the controlling shareholders of listed companies in the pharmaceutical manufacturing industry have equity pledge behaviors, indicating that the phenomenon of controlling shareholders in listed companies in the pharmaceutical manufacturing industry carrying out equity pledge is relatively common; among the control variables, the mean value of the corporate debt ratio (Lev) is 0.291, and the standard deviation is 0.151, indicating that the listed companies have innovative behaviors. is 0.151, indicating that listed companies are generally operating in debt; the standard deviation of company size (size) is 0.938, and the minimum and maximum values are 23.774 and 20.398, respectively, indicating that the size of each listed company is relatively balanced in distribution although there are obvious differences. The mean value of the company’s growth capacity (growth) is 0.123, the maximum value is 0.695, and the minimum value is −0.251, indicating that the growth capacity of different listed companies in the pharmaceutical manufacturing industry varies significantly.

**Table 2:** Descriptive statistics.

Variable	*N*	Mean	p50	SD	Min	Max.
rd	1852	6.817	4.965	5.423	1.28	23.14
ple	1852	9.367	15.443	9.147	0	19.781
mo	1852	0.081	0.006	0.128	0	0.409
Kz	1852	−0.220	0.114	2.622	−12.563	8.419
age	1852	18.992	18.33	2.678	14.75	25.17
size	1852	21.972	21.945	0.938	20.398	23.774
roa	1852	0.062	0.059	0.06	−0.072	0.184
growth	1852	0.123	0.091	0.224	−0.251	0.695
lev	1852	0.291	0.269	0.151	0.077	0.595

### 4.2 Correlation analysis

The correlation coefficient between the controlling shareholder’s equity pledge (Ple) and the enterprise’s innovation input (RD) is significantly negative at the 1% level, which preliminarily verifies that H1 is valid, i.e., the behavior of controlling shareholder’s equity pledge inhibits the enterprise’s innovation input, which is not conducive to the enterprise’s innovation activities. The coefficient of the controlling shareholder’s equity pledge (Ple) and executive shareholding (mo) is 0.070, which is significantly positive at the 1% level, indicating that the changes in the internal and external environments of the enterprise caused by the controlling shareholder’s equity pledge behavior will promote the enterprise’s equity incentives for executives. The coefficient of executive shareholding (mo) and corporate innovation investment (RD) is 0.065, which is significantly positive at the 1% level, indicating that risk-preferring managers will enhance corporate innovation investment, i.e., equity incentives are positively correlated with corporate innovation investment, and the preliminary validation of H2 holds. The correlation coefficients of other variables are below 0.7, indicating that there is no multicollinearity problem. In addition, the maximum value of the variance inflation factor VIF of one of the independent variables, 1.11, is much smaller than 10, indicating that there is no serious multicollinearity problem among the variables (Tables 3, 4).

**Table 3:** Correlation analysis.

	rd	ple	mo	Kz	age	size	roa
rd	1.000						
ple	−0.118***	1.000					
mo	0.065***	0.070***	1.000				
Kz	0.054**	0.152***	0.003	1.000			
age	0.069***	−0.076***	−0.097***	0.111	1.000		
size	−0.074***	0.073***	−0.223***	0.080***	0.092	1.000	
roa	−0.355***	−0.039*	0.162***	0.019	−0.071***	0.036	1.000
growth	0.121***	−0.031	0.111***	0.053**	−0.015	0.007	0.185
lev	−0.043*	0.109***	−0.097***	−0.057**	0.076***	0.250***	−0.359***
	growth	lev					
growth	1.000						
lev	−0.004	1.000					

**Table 4:** Multiple covariance analysis.

Variable	VIF	1/VIF
lev	1.250	0.798
roa	1.210	0.829
size	1.100	0.911
growth	1.040	0.966
ple	1.030	0.975
age	1.020	0.976
Mean VIF	1.110	

### 4.3 Basic regression analysis

#### 4.3.1 Impact of controlling shareholders’ equity pledge on firms’ innovation investment

In order to verify the role of controlling shareholders’ equity pledge on corporate innovation investment, regression test is conducted on model (1), and the test results are shown in Table 5. The result (1) does not contain control variables, and the result (2) introduces each control variable. From Table 5, regardless of the existence of control variables, the effect of controlling shareholders’ equity pledge on enterprises’ innovation investment is negative and significant, indicating that controlling shareholders’ equity pledge does inhibit enterprises’ innovation investment. In Table 5 (2), the correlation coefficient between controlling shareholders’ equity pledge (Ple) and firms’ innovation investment (rd) is −0.221, which is significantly negative at the 1% level, indicating that controlling shareholders’ equity pledge behavior inhibits firms’ innovation investment and is not conducive to firms’ innovation projects.

**Table 5:** Benchmark regression analysis.

	(1)	(2)
	ols1	ols2
ple	−0.218^***^	−0.221^***^
	[0.0586]	[0.0559]
age		0.324
		[0.3973]
size		−0.749
		[0.7518]
roa		−174.4^***^
		[28.2592]
growth		10.93^***^
		[3.1162]
lev		−31.69^***^
		[7.9122]
_cons	12.55^***^	40.89^*^
	[1.1898]	[15.9076]
*N*	1852	1852
adj. *R*^2^	0.0595	0.2352
*AIC*	17780.7	17402.6
*BIC*	17791.7	17441.3

Standard errors in brackets.

^*^p < 0.05, ^**^p < 0.01, ^***^p < 0.001.

Controlling shareholders will use their decision-making power to reduce the investment in innovation activities of the enterprise in order to avoid the loss of control. After the equity pledge, the controlling shareholders will face the risk of loss of control so they have to maintain the stability of the stock price and reduce the investment in R&D. Moreover, the equity pledge will lead to the loss of some of the cash flow rights, so in order to make up for the loss, the controlling shareholders will reduce the investment in R&D, which infringes on the interests of small and medium-sized shareholders. So the controlling shareholders will adopt to reduce the R&D investment decision. Moreover, the equity pledge increases the risk of failure of R&D investment, and the executives will adopt the reduction of R&D investment program if they think that the return of R&D investment is less than the risk.

#### 4.3.2 Controlling shareholders’ equity pledges, equity incentives, and corporate innovation investment

From the regression results of Equation 1 in Table 6, the regression coefficient between controlling shareholders’ equity pledge (Ple) and firms’ innovation investment (RD) is −0.221, which is significantly negative at the 1% level, indicating that controlling shareholders’ equity pledging behavior does inhibit firms’ innovation investment. From the regression results of Equation 2, the correlation coefficient between controlling shareholders’ equity pledge (Ple) and equity incentives (mo) is 0.00158, which is significantly positive at the 1% level, indicating that controlling shareholders’ equity pledge promotes equity incentives. From the regression results of Equation 3, controlling shareholders’ equity pledge (Ple) is also negatively correlated with corporate innovation investment (RD) with a correlation coefficient of −0.266, and the correlation coefficient is still significant at the 1% level. And the mediating variable equity incentive (mo) is positively correlated with corporate innovation investment (RD) at 1% significance level, with a correlation coefficient of 28.43, indicating that the mediating variable plays a very important role. At the same time, combining the regression results of Equations 1, 2, it can be concluded that equity incentives play a partly mediating role in the impact of controlling shareholders’ equity pledge on corporate innovation investment, and H2 passes the test and the hypothesis is established.

**Table 6:** Mediated effects test.

	Rd	Mo	Rd
ple	−0.221^***^	0.00158^***^	−0.266^***^
	[0.0559]	[0.0003]	[0.0602]
mo			28.43^***^
			[7.6980]
age	0.324	−0.00490^***^	0.464
	[0.3973]	[0.0013]	[0.4081]
size	−0.749	−0.0324^***^	0.171
	[0.7518]	[0.0031]	[0.7535]
roa	−174.4^***^	0.268^***^	−182.0^***^
	[28.2592]	[0.0441]	[28.8820]
growth	10.93^***^	0.0207^***^	10.34^***^
	[3.1162]	[0.0060]	[3.1099]
lev	−31.69^***^	−0.0121	−31.35^***^
	[7.9122]	[0.0207]	[7.8118]
mo			28.43^***^
			[7.6980]
_cons	40.89^*^	0.853^***^	16.64
	[15.9076]	[0.0697]	[16.7721]
*N*	1852	1852	1852
adj. *R*^2^	0.2352	0.1791	0.2463
*AIC*	17402.6	−2752.1	17376.3
*BIC*	17441.3	−2713.4	17420.5

Standard errors in brackets.

^*^p < 0.05, ^**^p < 0.01, ^***^p < 0.001.

By synthesizing the regression results of Equations 1–3 in Table 6, it shows that equity incentives (MO) are indeed a risk-transfer mechanism for controlling shareholders to inhibit firms’ innovation investment. That is, conducting executive equity incentives suppresses the negative effect of controlling shareholders’ equity pledge rate on R&D investment. If the equity incentive makes the return on R&D investment greater than the risk of failure, executives will choose to increase R&D investment, and this is when executives will monitor the controlling shareholders’ behavior at the expense of small and medium-sized shareholders in order to protect their own future interests and will not collude with the controlling shareholders and will increase the level of R&D investment.

#### 4.3.3 Controlling shareholders’ equity pledge, financing constraints, and corporate innovation investment

Table 7 describes the regression results of Model 4 with the addition of Pledge*kz, which represents the cross-multiplication term of controlling shareholders’ equity pledge rate and financing constraints. As can be seen from Table 7, the impact of controlling shareholders’ equity pledge on firms’ R&D investment is significant at the 1% level, and the coefficient of equity pledge is −0.177. It indicates that the controlling shareholders’ equity pledge rate inhibits firms’ R&D investment. The coefficient of the cross-multiplier term (Pledge*kz) is −1.454, and it is significantly negatively correlated at the 10% level, and the first column shows that equity incentives have a negative relationship with R&D investment. In summary, financing constraint behavior promotes the negative effect of controlling shareholders’ equity pledge ratio on R&D investment.

**Table 7:** Moderating effects test.

	Rd	Rd
ple		−0.177^***^
		[0.0517]
kz	−1.454^*^	
	[0.5710]	
Kz*ple		−0.0918^***^
		[0.0247]
age	0.403	0.377
	[0.4018]	[0.3970]
size	−0.987	−0.873
	[0.7449]	[0.7513]
roa	−192.3^***^	−193.2^***^
	[33.0849]	[32.9021]
growth	10.46^***^	10.44^***^
	[3.1269]	[3.0721]
lev	−21.35^*^	−20.55^*^
	[8.3174]	[8.3266]
_cons	40.67^**^	40.46^**^
	[15.4704]	[15.4393]
*N*	1852	1852
adj. *R*^2^	0.2387	0.2451
*AIC*	17394.0	17380.4
*BIC*	17432.6	17430.1

Standard errors in brackets.

^*^p < 0.05, ^**^p < 0.01, ^***^p < 0.001.

## 5 Heterogeneity analysis

### 5.1 Tests for heterogeneity in the nature of property rights

The State owns the property rights of SOEs, which are also subject to additional legal limitations and are overseen at all levels by the State-owned Assets Supervision and Administration Commission. In comparison to non-state listed enterprises, SOEs are subject to stricter supervision. Following the pledge of equity in state-owned enterprises, R&D investment is less likely to be impacted by controlling shareholders’ opportunistic actions to harm small and medium-sized shareholders or to prevent the transfer of control to stabilize the stock price behavior. This is because their conspiracy with executives will be subject to more stringent supervision (31). Because the state will assume the risk of failure and because SOEs will receive more sufficient government support and cash flow, the damage to SOEs should an investment fail is minimal. However, the liability of non-SOEs for their own losses is far weaker than that of SOEs. The property rights of SOEs belong to the State, are supervised by the State-owned Assets Supervision and Administration Commission (SASAC) at all levels, and are subject to more legal restrictions. The supervision of SOEs is more severe than that of non-state listed companies. After the pledge of equity in state-owned enterprises, for the controlling shareholders regardless of its damage to small and medium-sized shareholders of opportunistic behavior or in order to prevent the transfer of control to stabilize the stock price behavior, when it is colluding with the executives of the act will be subjected to more stringent supervision, the equity pledge is unlikely to R & D investment impact. Once the investment fails, the blow to SOEs is relatively small, as the state will bear the risk of failure and SOEs will receive more adequate government support and cash flow. But non-SOEs are responsible for their own losses, which is completely weaker than SOEs. Li noted that, in contrast to state-owned enterprises, non-state-owned enterprises rely on self-financing and place greater emphasis on risk management to prevent business failure and ensure long-term development (32). Consequently, to foster company development and mitigate the risk of business failure, risk management will increasingly be crucial for non-state-owned enterprises, potentially leading to a reduction in R&D investment. Consequently, following the equity pledge, non-state-owned enterprises require additional equity incentives to enhance R&D investment. Therefore, this paper carries out the analysis after distinguishing the nature of property rights, as the results in Table 8 show that in the non-state-owned sample, the coefficient of equity pledge is −0.167 and is significant at the 5% level. In the sample of state-owned enterprises, the coefficient of equity incentives is not significant. Therefore, the negative relationship between equity incentives suppressing controlling shareholders’ equity pledge and R&D investment is more significant in non-state-owned firms than in state-owned firms.

**Table 8:** Heterogeneity analysis.

	Government owned	Non-municipal	Lower shareholding concentration	Higher shareholding concentration
	Rd	Rd	Rd	Rd
ple	0.0567	−0.167^**^	−0.711^**^	−0.176^***^
	[0.0377]	[0.0612]	[0.3485]	[0.0544]
age	−0.214^*^	0.443	2.887^*^	−0.215
	[0.0942]	[0.2900]	[1.2065]	[0.4108]
size	−0.802^***^	1.031^*^	2.931	−0.784
	[0.2235]	[0.4930]	[2.8793]	[0.8737]
roa	−8.633^*^	−123.4^***^	−225.8^***^	−163.5^***^
	[4.3021]	[32.3951]	[67.1665]	[31.4845]
growth	−0.193	8.584^*^	7.258	12.37^**^
	[0.6115]	[3.4928]	[3.7329]	[3.9605]
lev	−4.946^**^	−34.91^***^	−49.41^*^	−27.03^**^
	[1.6668]	[8.7889]	[23.9308]	[8.5367]
_cons	28.39^***^	−3.778	−72.67	48.70^**^
	[4.9438]	[9.8251]	[61.9460]	[17.9905]
*N*	260	1231	323	1526
adj. *R*^2^	0.3020	0.1965	0.2053	0.2438
*AIC*	1259.5	10961.7	3254.6	13934.0
*BIC*	1284.4	10997.5	3281.0	13971.3

Standard errors in brackets.

^*^p < 0.05, ^**^p < 0.01, ^***^p < 0.001.

### 5.2 Heterogeneity test for equity concentration

Over-concentration of stock influences the enterprise’s decision-making mode and amplifies the profit-seeking behavior of controlling shareholders. As a result, in companies with more stock concentration, controlling shareholders have greater incentives to ‘hollow out’ (33). In order to test the effect of heterogeneity of equity concentration, this paper divides the sample into two groups of higher and lower equity concentration, and the results of the group test are shown in columns (3, 4) of Table 8. In the group with higher equity concentration, the coefficient of controlling shareholders’ equity pledge (Ple) is −0.176 and significant at 1% level, while in the group with lower equity concentration, the coefficient of Pleg is −0.711 and significant at 5% level. This indicates that the inhibitory effect of controlling shareholders on R&D investment is more serious in firms with higher equity concentration.

## 6 Endogeneity test and robustness test

This research performs endogeneity and robustness tests to improve the validity of the empirical results and to prevent endogeneity issues.

### 6.1 Endogeneity test

In order to eliminate the possibility of the existence of endogeneity problem, this paper adopts the instrumental variable method to test it. Combined with the specificity of equity pledge, this paper refers to the research of Xie Deren (34), and takes the lagged one period of equity pledge ratio of other firms in the same province in the same year (L.ple), the average of the annual equity pledge ratio of other firms in the same year (e_ple1), and the average of the annual equity pledge ratio of other firms in the same province (e_ple2) as instrumental variables of the equity pledge ratio (ple) respectively, and the results are shown in Table 9. The results are shown in Table 9. The first-stage regression results show that the coefficients of the three instrumental variables are positive and passed the significance level test. The results of weak instrumental variable test show that the *F*-value is 420.4, 22.13, 78.72, which is much larger than 10, indicating that the lagged one period of the equity pledge ratio of other enterprises in the same province in the same year (L.ple), the average of the annual equity pledge ratio of other enterprises in the same year (e_ple1), and the average of the annual equity pledge ratio of other enterprises in the same province (e_ple2) are used as the instruments of equity pledge ratio (ple). Proportion (ple) instrumental variables are suitable for the robustness test of two-stage least squares and the instrumental variables meet the requirements. The results of the second-stage regression show that both equity pledges and corporate innovation are significantly negatively correlated at the 1% level, and H1 still holds. This indicates that controlling shareholders’ equity pledging behavior negatively affects firms’ investment in R&D and innovation and the conclusion is robust.

**Table 9:** Endogeneity test.

	(1)	(2)	(1)	(2)	(1)	(2)
Variables	ple	rd	ple	rd	ple	rd
L.ple	0.620***	−0.173*				
	(0.020)	(0.103)				
e_ple1			0.834***	−2.580***		
			(0.177)	(0.762)		
e_ple2					0.974***	−1.080***
					(0.109)	(0.329)
Observations	1,537	1,537	1,852	1,852	1,852	1,852
R-squared		0.132		−0.244		0.151
Firm FE	Yes	Yes	Yes	Yes	Yes	Yes
Year FE	Yes	Yes	Yes	Yes	Yes	Yes
rkf	420.4	420.4	22.13	22.13	78.72	78.72

Standard errors in brackets.

^*^p < 0.05, ^**^p < 0.01, ^***^p < 0.001.

### 6.2 Regression using one period lagged data

From Table 10, it can be found that when lagging firms’ breakthrough innovations by two orders, controlling shareholders’ equity pledges still significantly inhibit firms’ breakthrough innovations at the 1% level, and hypothesis H1 holds. This result also further shows that the impact of firms’ breakthrough innovation is long-term rather than short-term.

**Table 10:** Robustness test.

	Rd	L.Rd	Rd
ple	−0.221^***^	−0.124^**^	
	[0.0559]	[0.0497]	
Ple_1			−0.622^***^
			[0.1278]
age	0.324	0.675^*^	0.414
	[0.3973]	[0.2992]	[0.4017]
size	−0.749	−0.369	−1.013
	[0.7518]	[0.5296]	[0.7550]
roa	−174.4^***^	−126.2^***^	−174.9^***^
	[28.2592]	[27.5593]	[28.3032]
growth	10.93^***^	18.64^***^	11.23^***^
	[3.1162]	[5.3533]	[3.1252]
lev	−31.69^***^	−27.68^***^	−32.07^***^
	[7.9122]	[6.1920]	[7.9477]
_cons	40.89^*^	17.68	44.21^**^
	[15.9076]	[12.0032]	[16.0270]
*N*	1852	1537	1852
adj. *R*^2^	0.2352	0.2175	0.2361
*AIC*	17402.6	13885.1	17400.3
*BIC*	17441.3	13922.4	17439.0

Standard errors in brackets.

^*^p < 0.05, ^**^p < 0.01, ^***^p < 0.001.

### 6.3 Substitution of explanatory variables

Referring to the practice of scholars such as Xie Deren and Tang Wei, the explanatory variable controlling shareholders’ equity pledge measure is replaced. In this paper, the number of shares pledged by controlling shareholders’ equity accounts for the ratio of the number of shares pledged by controlling shareholders to the total share capital of the company at the end of the year (Ple_1) is used as a proxy variable for controlling shareholders’ equity pledge to conduct a robustness test, as shown in Table 10, and the significance of the control variables and the positive and negative values of the regression coefficients are basically the same as those of Table 10, which leads to the conclusion that the empirical analyses of the previous paper are robust, and that the inhibitory effect of equity pledge on R&D of enterprises is reliable. The conclusion of the role of equity pledge is reliable.

## 7 Conclusion and recommendations of the study

### 7.1 Conclusion of the study

This paper examines the current state of controlling shareholders’ equity pledges in China’s A-share market, analyzing the correlation between these pledges and innovation investment in pharmaceutical manufacturing firms. It investigates the influence of equity incentives on both controlling shareholders’ equity pledges and corporate innovation investment, along with their transmission mechanisms, while also exploring the moderating effect of financing constraints. The results are as follows: (1) Controlling shareholders’ equity pledge behavior inhibits firms’ innovation input. From the standpoint of financial constraints and self-interested control, despite the substantial advantages that successful innovation activities can yield for an enterprise’s future growth, the inherent risks associated with innovation investments and the delayed realization of benefits imply that, should an innovation fail, investors are likely to liquidate significant portions of their shares in the secondary market, thereby precipitating a risk of share price collapse. If the stock price declines to the ‘warning line’ or ‘close-out line’ and the controlling shareholder is unable to provide additional margin or collateral, the pledged shares may be forcibly liquidated. To prevent loss of control, the controlling shareholder will curtail the innovation project to ensure adequate cash flow for the company. The organization possesses adequate cash flow. (2) Equity incentives play a partly intermediary role in the process of controlling shareholders’ equity pledge inhibiting firms’ innovation investment. When the controlling shareholder pledges equity in order to reduce share price volatility, it will use equity incentives to the executives, so that the executives will focus on the operation of the enterprise and release positive signals to the outside, thereby reducing share price volatility. Thus, equity incentives play a partially mediating role between controlling shareholders’ equity pledges and firms’ innovation investment. (3) Financing constraint behavior promotes the negative impact of equity pledges on corporate innovation. The capital market will receive a signal that the enterprise’s financial chain is broken when shareholders’ equity pledges are controlled. Large enterprises are particularly susceptible to media attention and tracking reports, which leads to an amplification effect that further exacerbates the deterioration of the enterprise’s financing environment. The high dependence of enterprise innovation activities on capital is in stark contrast to the financial difficulties that enterprises are currently facing, which inherently discourages them from investing in innovation.

### 7.2 Policy recommendations

- This paper will present pertinent recommendations for both government and enterprises based on the research conclusions outlined above. Firstly, government departments should enhance the laws and regulations governing controlling shareholders’ equity pledges, while relevant authorities must intensify the qualification assessment and oversight of controlling shareholders’ equity pledging activities. In recent years, regulators have implemented additional constraints and requirements regarding the equity pledges of controlling shareholders; however, a more detailed explanation of the utilization of the pledged funds is also necessary. Due to an inadequate policy framework and insufficient oversight, certain pledged controlling shareholders may circumvent their disclosure responsibilities, misappropriate pledged funds, and participate in insider trading, contravening legal regulations. The market environment for equity pledge financing deteriorates, leading to a decline in investor trust in the company, thereby posing significant challenges to the company’s innovative development (35, 36). To mitigate financing risks associated with equity pledges and foster the healthy development of this market, financial institutions ought to elevate the threshold for controlling shareholders’ equity pledges and implement rigorous audits of the qualifications of financing companies. Concurrently, relevant supervisory authorities should enhance oversight throughout the transaction process and the utilization of subsequent funds, refine applicable laws and regulations, and render judgments based on various specific circumstances. The actions of controlling shareholders require stringent regulation to mitigate financial systemic risks.
- Second, for enterprises, financing received by listed companies in the pharmaceutical manufacturing industry through stock pledges has a negative impact on the enterprise’s innovation investment, which helps to strengthen the enterprise’s long-term competitiveness. In the short term, equity pledge enterprises may be exposed to the risk of a strong flat or supplemental margin in the presence of capital market turbulence. However, the probability of controlling shareholders’ equity pledges occurring closeout or supplemental margin is exceedingly low under the current system of China’s Measures. Therefore, pharmaceutical manufacturing companies should be adept at utilizing capital market equity pledge financing methods to enhance their core competitiveness through innovation in research and development investment, thereby fostering the sustainable and healthy development of enterprises. We should enhance marketization efforts, improve the multi-level structure of the capital market, expand both direct and indirect financing channels for the pharmaceutical manufacturing sector, and facilitate financing conditions for small and medium-sized enterprises in China. Consequently, the acceleration of marketisation is necessary to diminish enterprises’ reliance on government and to establish a competitive market environment that is fair and open. Listed companies in the pharmaceutical manufacturing sector must focus on their ownership structure to mitigate the risks of major shareholders engaging in ‘hollowing out’ and minor shareholders ‘hitching a ride.’ Additionally, it is essential to broaden financing channels, enhance direct financing guidance, and adhere to the principle of incentive compatibility to promote increased investment in innovation. Promote the enhancement of enterprise investment in innovation (37, 38).

## Data Availability

The original contributions presented in the study are included in the article/supplementary material, further inquiries can be directed to the corresponding authors.
